# Advancing Nutritional Status Classification With Hybrid Artificial Intelligence: A Novel Methodological Approach

**DOI:** 10.1002/brb3.70548

**Published:** 2025-05-13

**Authors:** Md. Moddassir Alam, Asif Irshad Khan, Aasim Zafar, Mohammad Sohail, Mohammad Tauheed Ahmad, Rezaul Azim

**Affiliations:** ^1^ Department of Health Information Management and Technology, College of Applied Medical Sciences University of Hafr Al Batin Hafr Al Batin Saudi Arabia; ^2^ Department of Computer Science Aligarh Muslim University Aligarh India; ^3^ Data Architect and AI/ML Engineer, Hewlett Packard Enterprise Pennsylvania USA; ^4^ College of Medicine King Khalid University Abha Saudi Arabia; ^5^ Faculty of Science University of Chittagong Chittagong Bangladesh

**Keywords:** child malnutrition, children, clustering analysis, hybrid AI strategies, hybrid model, machine learning in nutrition, malnutrition detection, nutritional status analysis

## Abstract

**Purpose:**

Malnutrition remains a critical public health issue in low‐income countries, significantly hindering economic development and contributing to over 50% of infant deaths. Under nutrition weakens immune systems, increasing susceptibility to common illnesses and prolonging recovery periods. This study aims to develop and evaluate a novel artificial intelligence‐based classification method for nutritional status assessment using hybrid machine learning strategies, enhancing the accuracy and reliability of malnutrition detection.

**Method:**

This study utilizes the fire hawk optimizer‐based k‐means (FHO‐K‐Means) clustering method to identify key physiological indicators associated with under nutrition in children. The analysis is conducted using the UNICEF dataset, encompassing data from Afghanistan, Albania, Algeria, and Zimbabwe. Following data normalization, the dataset is clustered via FHO‐K‐Means to establish optimal groupings. The clustered data is then partitioned into training and testing sets for classification using the extreme gradient boosting fuzzy (EGBF). The EGBF model is employed to classify nutritional states, including stunting, wasting, severe wasting, overweight, and underweight, providing a robust framework for predictive analysis.

**Findings:**

The proposed FHO‐K‐Means and EGBF model demonstrated superior performance, achieving 99.84% accuracy, 99.5% precision, 99.8% specificity, and 100% sensitivity, with an F1 measure of 98.6% and a mean squared error (MSE) of 0.01%, outperforming existing classification techniques. These results indicate that the model offers a highly effective and scalable tool for identifying at‐risk populations, informing targeted interventions to reduce the prevalence of childhood malnutrition, and lowering morbidity in poor countries.

**Conclusion:**

This study developed an innovative FHO‐K mean clustering and EGBF classification method for assessing childhood nutritional status in underdeveloped countries. The exceptional accuracy and predictive capability of the model make it a valuable tool for early malnutrition detection, enabling data‐driven decision‐making in public health and policy formulation. By improving malnutrition diagnosis and intervention strategies, this approach has the potential to reduce morbidity and enhance child health outcomes in resource‐constrained settings.

## Graphical abstract

This graphical abstract illustrates a novel hybrid AI framework that integrates Fire Hawk Optimizer‐based K‐Means clustering with Extreme Gradient Boosting Fuzzy classification to accurately identify and classify childhood nutritional statuses, enabling data‐driven interventions for malnutrition in developing nations.

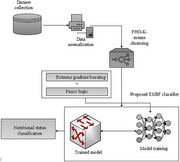

## 1 Introduction

Malnutrition in children is still a major problem in many poor nations, while being rare in industrialized nations (Sieber 2019). In many nations across the world, childhood under nutrition, especially among children under the age of five, is a serious health issue that harms children's development. According to the world health organization (WHO), in 2020, there were 149 million stunted children (those who are abnormally little for their gestational age), 45 million wasted children (those who are too small for their growth), and 38.9 million overweight children (Lall et al. 2022).

Malnutrition in children is characterized by one or more of stunting, underweight, and wasting. Malnutrition comes in many forms, including under nutrition, malnutrition, and over nutrition, linked to micronutrients (Calcaterra and Zuccotti 2022). Children who exhibit signs of malnutrition (wasting, stunting, and underweight) have a higher risk of illness and mortality. The incidence of malnutrition is still concerning because stunting is reducing inadequately while wasting continues to harm the growth of too many kids under the age of five (Hsu et al. 2023). Short height for age is a sign of stunting; as is low weight for age, and low weight relative to height is a sign of wasting. Iron deficiency is a kind of malnutrition associated with micronutrients. Obesity and overweight are signs of overeating and risk factors for non‐communicable diseases (NCDs) (Dang 2023). The worldwide incidence of underweight and stunting was estimated by the world bank, WHO, and United Nations children's fund (UNICEF) in 1990 (Derakhshandeh‐Rishehri et al. 2023). The study also found that in countries with low‐to‐middle‐incomes, under nutrition is a factor in about 45% of the mortality of children under the age of five. Children in underdeveloped nations continue to experience the majority of illnesses, premature deaths and mortality due to under nutrition (Ibrahim et al. 2022; Sotiraki et al. 2022).

Half of all children under the age of five die from causes related to undernourishment; this condition increases the risk of common diseases, makes them more severe and frequent, and hinders their recovery time (Larson‐Nath and Goday 2019). Therefore, improving malnutrition early detection and treatment will be necessary to achieve additional meaningful decreases in under‐five mortality. Accurate weight and height measurements may be difficult in areas with a lack of resources when malnutrition is widespread (House and Gwaltney 2022) (Compher et al. 2022). The integrated handling of childhood diseases encourages healthcare professionals to evaluate the nutritional status of each presenting child. All levels of under nutrition have a detrimental influence on health (Wells et al. 2019). A chance to stop long‐term morbidity from affecting a person's quality of existence, growth, academic success, and future employment opportunities is lost if under nutrition is not identified.

According to traditional methods of identifying causes of mortality, immunizable illnesses, severe respiratory infections, diarrhea, and malaria account for around 70% of all child fatalities (aged 0–4 years) globally (Ekholuenetale et al. 2020). Despite the centuries‐old understanding of the cooperation between hunger and infectious illnesses, the significance of malnutrition in mortality among children is not shown by these traditional approaches (Djoumessi 2022). Computer programs may learn from databases that show situations and knowledge, detect chemicals, and assist in decision‐making by addressing difficulties thanks to artificial intelligence (AI) (Raphaeli and Singer 2021; Toffaha et al. 2023; Gadekallu et al. 2021; Najaflou and Rabiei 2021). There are several uses for AI, notably in the delivery of nutrition and health care. In the evaluation of individualized nutritional guidance and meal schedules that can enhance patients' meal and nutrient intake, AI can play a major role in personalized nutrition. Additionally, it can spot people who are at risk for malnutrition and offer suggestions for improving nutritional status (Momand et al. 2022; Shi et al. 2022).

The current paper's goal was to investigate the effects of this policy change as they pertain to the single result of young child survival. The goal was to specifically look at the association between fluctuations in infant and under‐five death rates in countries that have been developing over the past two to three years, and shifts in children's overall nutritional condition over the same period. Moreover, this study identifies critical physiological indicators of under nutrition in children using an innovative FHO‐K‐Means clustering method, leveraging the comprehensive UNICEF dataset covering 74 countries, including Afghanistan, Albania, Algeria, and Zimbabwe. The primary intention is to predict child nutritional status through a multifaceted approach involving data normalization, clustering, and classification. Initially, the dataset undergoes normalization, followed by clustering via FHO‐K‐means, dividing it into optimal groups. The clustered data is then split into training and testing sets for EGBF Classification. This EGBF approach categorizes nutritional states into stunting, wasting, severe wasting, overweight, and underweight, enabling targeted interventions. By integrating FHO‐K‐means clustering and EGBF classification, the study evaluates the performance of this combined approach, demonstrating its potential to inform evidence‐based policy decisions and interventions aimed at mitigating child malnutrition. Ultimately, the research seeks to provide actionable insights for healthcare professionals, policymakers, and stakeholders to address under nutrition effectively.

The main contribution of this study is explained below:

· The study examines aggregate‐level data on pediatric malnutrition and mortality across developing nations, utilizing data sourced from the Kaggle platform.
· The nutritional data from the dataset is analyzed using the FHO‐K‐Means clustering approach to accurately classify malnutrition types. In this method, the K‐Means algorithm is employed for clustering, while the fire hawk optimizer (FHO) enhances its performance by optimizing cluster formation, improving classification accuracy, and convergence efficiency.
· The study introduces EGBF classification, an enhancement over traditional machine learning techniques, to improve the accuracy of malnutrition classification.
· The performance of the proposed model is evaluated using various metrics such as accuracy, sensitivity, specificity, MSE, precision, and F1 measure, demonstrating its superior performance over existing classification techniques.

The remainder of this paper is structured as follows: Section 2 presents a literature review of recent studies on malnutrition assessment. Section 3 details the proposed methodology and its mathematical foundation. Section 4 discusses the results and comparative analysis, while Section 5 concludes the study with findings, limitations, and future research directions.

## 2 Related Work

To tackle malnutrition effectively, it is essential to use thorough systems for analyzing and categorizing nutritional status in order to precisely identify and provide intervention for groups at risk. Conventional methods for evaluating nutritional status typically use manual techniques that can be time‐consuming, subjective, and susceptible to mistakes. Recent breakthroughs in AI have provided intriguing potential to improve the precision, effectiveness, and scalability of nutritional status assessments. Mukuku et al. 2019 developed a prognostic score for severe acute malnutrition (SAM) in children under five years old. They conducted a case‐control study and carried out both multivariate and univariate analyzes. The discrimination score was assessed using the ROC curve and the Hosmer‐Lemeshow test for calibration. A straightforward and efficient prediction approach was suggested to estimate the probability of SAM incidence in children under five in developing countries. The SAM prediction model functioned effectively as a treatment tool to identify persons at risk, decrease malnutrition rates, and lower the occurrence of disease and infant death in undeveloped countries.

Ahmad et al. 2020 sought to determine how socioeconomic variables affected malnutrition in children under the age of five in Pakistan's Multan region, which is part of the Punjab state. Researchers examined responses from 2,497 kids who were part of the 2018 multiple integrated cluster survey to determine the real effect of socioeconomic factors on childhood malnutrition. People from lower socioeconomic origins, especially those living in rural regions, had a higher prevalence of malnutrition, according to the study.

In their work, Talukder and Ahammed 2020 used a variety of algorithms for machine learning (ML) to forecast the nutritional condition of under‐five‐year‐old children in Bangladesh. The 2014 Bangladesh demographic and health survey (BDHS) secondary records that were nationally representative were used for analysis. Support vector machines (SVM), logistic regression (LR), random forest (RF), linear discriminant analysis (LDA), and k‐nearest neighbors (k‐NN) are five well‐known ML techniques that have been explored to reliably predict the condition of malnutrition in children. Additionally, specificity, accuracy, sensitivity, and Cohen's statistics were used to conduct a thorough evaluation of the algorithms. Finally, the research suggests using RF classification with RF selection of features when the main goal is to predict malnutrition.

Yunus, R. M., et al. (Khan and Yunus 2023) utilized the under‐five nutritional secondary data from the sub‐Saharan African nations' Demographic and Health Surveys. The MVBHE model was created by the study using boosting, bagging, and voting algorithms such as RF, decision tree, extreme gradient boosting, and KNN machine learning techniques. The MVBHE model was then used to increase the accuracy to 96%. The MVBHE model is suggested by the current study as the most precise scheme for predicting undernourishment in kids under the age of five.

How and Chan 2020 study used a simple, easy Bayesian statistical modeling method based on artificial intelligence to show that probabilistic analysis centered around human factors could be employed to analyze the patterns of worldwide malnutrition and enhance circumstances to accomplish the ideal situation. Circumstances like the “Black Swan” situation were also modeled and might be utilized to warn participants to avoid them from occurring. As a result, vulnerable people's nutritional and physical conditions may be improved.

Data from the 2016 Ethiopian Demographics and Health Survey were utilized in the study by Takele et al. 2019. To determine demographic, socioeconomic, environmental, and health‐related risk variables for stunted children younger than five, a generalized linear mixed model variation on the general linear model was used. According to the research, children who are male, older (in the range of 12 to 59 months), underweight, not nursing, impoverished, living in homes without toilets, whose mothers are illiterate, and who have close childbearing intervals are all at risk for stunting. To solve the nation's difficulties with stunting in children under the age of five, education on family planning and policy is necessary.

Historical cross‐sectional survey results from Ethiopia, a nationally representative set of information gathered in various years, were used by Fenta et al. 2021 to apply ML algorithms. We looked at six popular machine learning (ML) techniques: neural network, elastic net, random forest (RF), LR with L‐2 regularization (ridge), and LR with L‐1 regularization. The best ML model was determined to be the RF algorithm based on several performance measurements.

A country‐by‐country analysis of the malnutrition rate, whether it has decreased or increased, is missing from standard publications. Additionally, the nations with the highest percentages of overweight, stunted, and wasted children under five by national income categorization and of all kinds of malnutrition were not verified.

### 2.1 Problem Statement

Existing research in nutritional status analysis primarily relies on conventional statistical methods or isolated AI techniques, such as machine learning or deep learning, which have inherent limitations in handling complex and heterogeneous nutritional data. These traditional approaches struggle to capture intricate relationships and patterns, leading to incomplete insights and potential biases. Furthermore, the focus tends to be narrow, concentrating on specific nutritional disorders or demographics, thereby neglecting the development of comprehensive and generalized frameworks for nutritional status analysis. This fragmented approach hinders the integration of diverse data sources and the consideration of multiple nutritional factors, resulting in a lack of standardization and scalability.

To address these gaps, there is a pressing need for innovative and adaptive methodologies that can effectively integrate multidimensional data, accommodate diverse populations, and provide personalized nutritional assessments. A holistic approach leveraging clustering and optimization algorithms, could potentially overcome existing limitations and contribute to the creation of robust, inclusive, and adaptable nutritional status analysis frameworks.

## 3 Proposed Methodology

**FIGURE 1:**
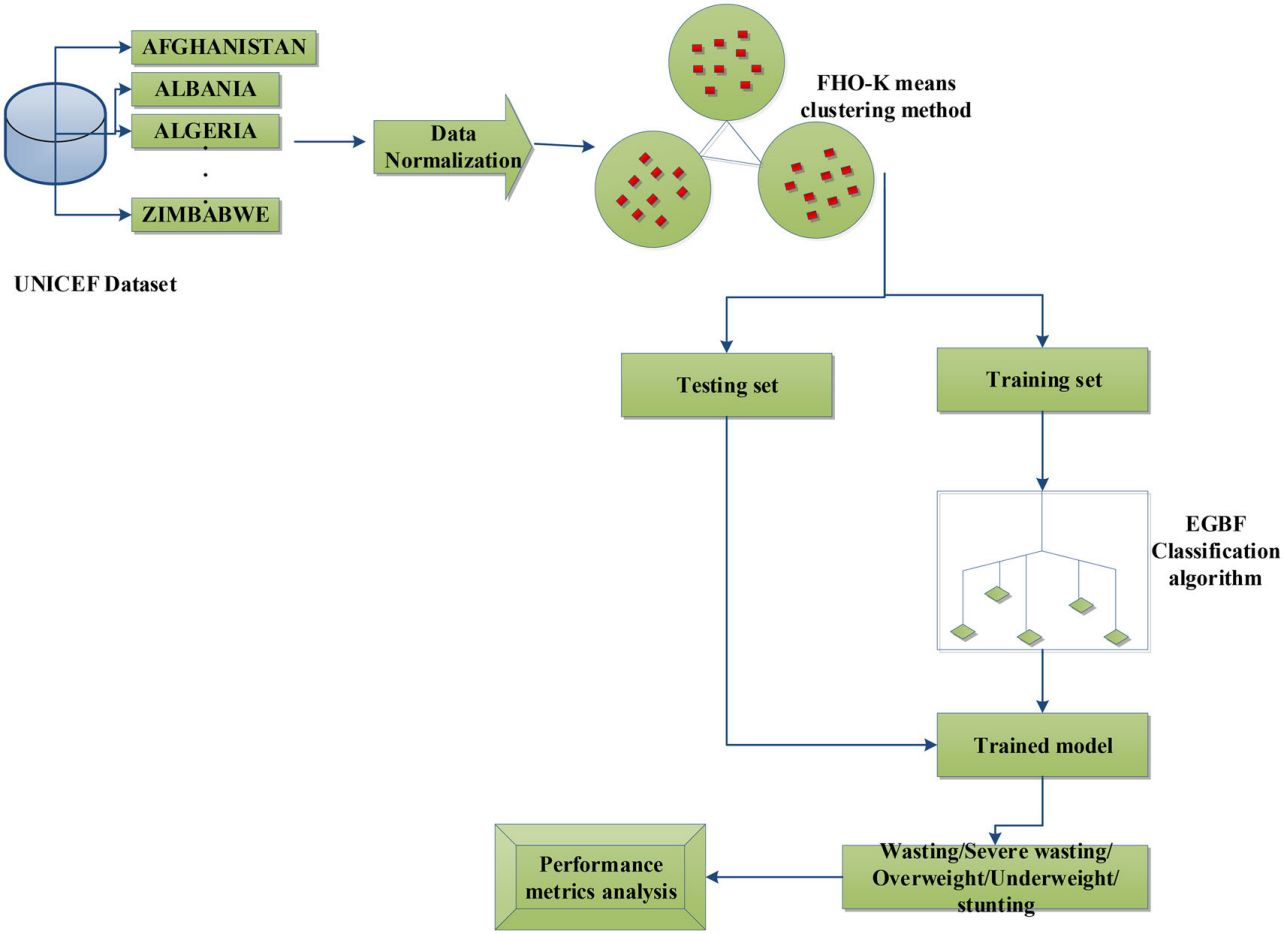
Proposed framework of nutritional status analysis.

The proposed framework of nutritional status analysis is illustrated in figure 1. The child malnutrition—UNICEF dataset from the kaggle website is to be collected for validation. More child data under the age of five can be included in this study to illustrate the nutritional condition of children. The classification and regulations might be based on dietary advice. The nutritional status of several developing nations is included in the dataset. Typically, raw data are not discovered in the format or form needed for the AI algorithms to function at their best. Therefore, in AI model applications, the pre‐processing phase is of utmost significance. For AI methods, the dataset is split at random into two portions: a training dataset that is employed to train the algorithm, and a test set used to forecast the outcome variable to assess the exactness of the forecasts against the real results. The validation dataset is applied for the estimation of parameters that will be included in the training approaches. To evaluate the performance of the computational models, in this research, 30% and 70% have divided the datasets into testing and training datasets. Consequently, the FHO‐K means clustering and EGBF was proposed for processing the database to find the nutritional status. The novelty of the FHO‐K means clustering lies in the seamless and effective integration of a metaheuristic optimizer named “FHO” and “k‐means clustering” into a single algorithm for clustering the nutritional dataset. This developed clustering algorithm identifies the patterns within the input database and groups them into different clusters, indicating different nutritional statuses like stunting, wasting, severe wasting, underweight, and overweight. This clustered nutritional database provides structured input for the proposed EGBF classifier, allowing it to classify the nutritional state of the children in real‐time scenarios with enhanced accuracy and less computational time. The EGBF was developed by combining the advantages and unique features of the extreme gradient boosting (XGBoost) approach and fuzzy logic for assessing nutritional status. The adaptive nature of fuzzy logic enables the system to handle uncertain data more effectively, boosting the system's adaptability to real‐world scenarios. On the other hand, the XGBoost model has the potential to process large datasets with high accuracy and better computational efficiency, which enhances the system's scalability and generalization efficiency. Thus, the combination of XGBoost and fuzzy logic into EGBF allows for processing the large uncertain nutritional data and classifies the nutritional status of children accurately with less computational time.

Here's a step‐by‐step explanation of the study:

Step 1: Data collection

The study uses the UNICEF dataset, which includes information from Afghanistan, Albania, Algeria, and Zimbabwe, to predict child nutritional status. The dataset contains information on various physiological indicators, such as height, weight, and age, for children under the age of five. The data were collected from various sources, including national surveys, health facilities, and community‐based programs.

Step 2: Data normalization

The collected data are normalized to ensure consistency and scalability, preparing it for clustering. Data normalization was performed to ensure that all features were on the same scale. This step is crucial in preventing features with large ranges from dominating the analysis. The min‐max normalization technique was used, which rescales numeric data to a common range between 0 and 1. The formula used for min‐max normalization is:

Step 3: Clustering using FHO‐K‐means

The normalized data were then clustered using the FHO‐K‐means algorithm, dividing the dataset into optimal groups. The FHO‐K‐means algorithm is a variant of the traditional k‐means clustering algorithm that uses the FHO to optimize the clustering process. The FHO is a nature‐inspired optimization algorithm that mimics the behavior of fireflies in search of food. The algorithm iteratively updates the cluster centers and assigns data points to the nearest cluster until convergence.

Step 4: Data split

The clustered data were split into training and testing sets for EGBF classification. The EGBF approach is a machine learning algorithm that combines multiple weak models to create a strong predictive model. The algorithm iteratively adds decision trees to the model, with each tree attempting to correct the errors of the previous tree. The EGBF approach was used to classify nutritional states, including stunting, wasting, severe wasting, overweight, and underweight.

Step 5: EGBF classification

The EGBF approach classifies nutritional states into five categories:

1. Stunting
2. Wasting
3. Severe wasting
4. Overweight
5. Underweight

Step 6: Performance analysis

The performance of the approach is evaluated, demonstrating its efficacy in predicting child nutritional status. The explanation of each step is given below.

### 3.1 Data Source

This research studied malnutrition disparities across 152 different countries. The dataset used for the analysis is Child Malnutrition—UNICEF Dataset 2024. The UNICEF child malnutrition dataset comprises 10,000+ observations with variables including age, sex, weight, height, mid‐upper arm circumference (MUAC), geographical location, and socioeconomic status. This dataset was employed to classify nutritional status using anthropometric measurements and demographic indicators, categorized into stunting, wasting, overweight, underweight, and severe wasting based on WHO child growth standards.

· Wasting is the percentage of children between 0 and 59 months old whose weight‐to‐height ratio is within plus or minus two standard deviations from the median.
· Severe wasting—percentage of infants and toddlers between 0 and 59 months who are under minus three standard deviations from the median body weight for height.
· Overweight ‐% of children aged 0 to 59 months with weight in relation to height discrepancies more than two standard errors.
· Stunting—Moderate and severe: % of infants and toddlers aged 0 to 59 months with heights less than two standard errors from the median for their age.
· Underweight—Moderate and severe: % of infants and toddlers aged 0 to 59 months who are under either one or two standard deviations from the median body weight for their age.

The dataset provides a comprehensive overview of the nutritional status of children in these countries, allowing for a thorough analysis of the association between fluctuations in infant and under‐five death rates and shifts in children's overall nutritional condition. The use of this dataset ensures that the findings of this study are generalizable to other developing countries. Furthermore, the dataset's large sample size provides sufficient statistical power to detect significant associations between variables.

### 3.2 Data Normalization

Data normalization is crucial for nutritional status analysis. It ensures that all variables are on the same scale. This prevents feature dominance and improves model performance. Normalization enhances interpretability and reduces the impact of outliers. Min‐max normalization is a popular technique used for nutritional status analysis. It rescales numeric data to a common range, usually between 0 and 1. Min‐max normalization is simple to implement and preserves relationships between features. It is robust to outliers and improves model performance. Min‐max normalization is computationally efficient and easy to interpret. It improves model stability and enhances data visualization. Normalization is essential for many machine learning algorithms. These algorithms are sensitive to the scale of the features. Normalization helps to improve model performance and prevent feature dominance. Min‐max normalization is a widely used technique in data science. It is a simple yet effective way to normalize data. Min‐max normalization is particularly useful for datasets with varying scales. It can handle categorical variables, making it a suitable choice for datasets with mixed variable types. Min‐max normalization is fast and efficient, making it suitable for large datasets. It is easy to implement and provides excellent results. Min‐max normalization is a reliable technique for nutritional status analysis. It provides accurate and reliable results, making it a popular choice among researchers. By using min‐max normalization, researchers can ensure that their data is normalized and ready for analysis. This helps to improve the accuracy and reliability of the results, making it a crucial step in nutritional status analysis. Using min‐max normalization, the range of each feature is normalized. This made it feasible to map every conceivable value for each property in the [0, (Sieber 2019)] range as well. To express the min‐max normalization, use Equation. (1).

(1) $$Y_{m,n}^{norm}=\frac{Y_{m,n}-\min\left(Y_{:,n}\right)}{\max\left(Y_{:,n}\right)-\min\left(Y_{:,n}\right)}$$

where $Y_{m,n}$ is the variable value of the attribute, is the value of the feature, and $\min(Y_{:,n})$ is the lowest value of the column feature; n=1,2,….j and j is the number of features; m=1,2,….i and i is the quantity of training or testing set data; and $\max(Y_{:,n})$ represents the column feature's maximum value. Scaling map components in the [0, (Sieber 2019)] range.

### 3.3 FHO‐K‐Means Clustering Approach

The FHO‐K‐Means clustering approach, which is employed to cluster the malnutrition values of various nations in the dataset, and combines metaheuristic and machine learning‐based AI methodologies (Azizi et al. 2023; Sinaga and Yang 2020). The fire hawk bird is the inspiration for this algorithm. Indigenous Australians utilized fire as a powerful weapon in their linguistic and ethnic customs to regulate and preserve the balance of the region's ecology and terrain. Human activity and other factors can make intentionally started fires, or those brought on naturally, including by lightning, worse, making the surrounding environment and wildlife more vulnerable. Black kites, whistling kites, and brown falcons have been identified as further contributors to the spread of wildfires in the area. Fire Hawks, a type of bird, are said to deliberately spread fire in the wild by carrying burning twigs in their beaks and talons. They use flaming branches to start tiny fires in unspoiled regions to control and trap their prey. Small flames like this startle prey such as mice, snakes, and various other creatures, forcing them to leave hastily and nervously, leading the hawks to capture them.

In addition, the fire hawk optimizer‐based k‐means clustering (FHO‐K‐Means) method is a powerful algorithm for nutritional status analysis. It is a variant of the traditional k‐means clustering algorithm that uses the Fire Hawk Optimizer (FHO) to optimize the clustering process. The FHO is a nature‐inspired optimization algorithm that mimics the behavior of fireflies in search of food. The algorithm iteratively updates the cluster centers and assigns data points to the nearest cluster until convergence. FHO‐K‐Means is particularly effective in handling high‐dimensional data and identifying complex patterns. It is a robust algorithm that can handle noisy and missing data. FHO‐K‐Means is also computationally efficient and can handle large datasets. The algorithm is easy to implement and provides accurate and reliable results. In nutritional status analysis, FHO‐K‐Means can be used to identify distinct clusters of children with similar nutritional profiles. This provides valuable insights into the relationships between nutritional indicators and mortality rates. FHO‐K‐Means can also be used to identify patterns and correlations between nutritional indicators and other factors such as age, sex, and socioeconomic status. The algorithm can handle multiple variables and provide a comprehensive understanding of the nutritional status of children. FHO‐K‐Means is a powerful tool for nutritional status analysis and can provide valuable insights for policymakers and healthcare professionals. By using FHO‐K‐Means, researchers can identify areas of high need and develop targeted interventions to improve the nutritional status of children. The algorithmic procedure is given below.

**Initialization**: The FHO metaheuristic approach models the foraging behavior of fire hawks by taking into account the creation and spread of fires as well as food collection. The location matrices of the fire hawks, as well as prey, are initially calculated from a number of response possibilities (Y). The starting locations of these variables in the search space are found by an arbitrary initialization method.

(2) $$Y=\left[\begin{array}{c} Y_{1}\\ Y_{2}\\ \vdots \\ Y_{j}\\ \vdots \\ Y_{n} \end{array}\right]=\left[\begin{array}{cccccc} Y_{1}^{1} & Y_{1}^{2} & \text{…} & Y_{1}^{k} & \text{…} & Y_{1}^{e}\\ Y_{2}^{1} & Y_{21}^{2} & \text{…} & Y_{2}^{k} & \text{…} & Y_{2}^{e}\\ & & \vdots \vdots \vdots & \ddots \vdots & & \\ Y_{j}^{1} & Y_{j}^{2} & \text{…} & Y_{j}^{k} & \text{…} & Y_{j}^{e}\\ & & \vdots \vdots \vdots & \ddots \vdots & & \\ Y_{n}^{1} & Y_{n}^{2} & \text{…} & Y_{n}^{k} & \text{…} & Y_{n}^{e} \end{array}\right],\;\;\;\left\{\begin{array}{c} j=1,2,\text{…}.n\\ k=1,2,\text{…}..e \end{array}\right.$$

(3) $$Y_{j}^{k}\left(0\right)=Y_{j,\min}^{k}+r.\left(Y_{j,\max}^{k}-Y_{j,\min}^{k}\right),\;\;\;\;\left\{\begin{array}{c} j=1,2,\text{…}.n\\ k=1,2,\text{…}..e \end{array}\right.$$

Where the $j^{th}$ contender for a solution in the search space is $Y_{j}$, e stands for the dimension of the issue under consideration. n is the number of potential solutions in the search space as a whole, $Y_{j}^{k}$ is the $j^{th}$ solution $k^{th}$ decision factor, $Y_{j}^{k}\left(0\right)$ shows the starting location of the potential solutions, $Y_{j,\max}^{k}$ and $Y_{j,\min}^{k}$ are the $j^{th}$ solution candidate's lowest and maximum boundaries for the $k^{th}$ decision variable. The objective function evaluates the solution candidates based on the specified optimization problem to determine the fire hawks' positions in the search space. Some k‐means solutions with higher objective function values are referred to as fire hawks, whereas the other options are considered prey. Fire hawks are selected to spread flames in the search area to make hunting easier. The fire hawks' initial usage of the primary fire to spread flames over the search zone is considered the most effective strategy.

(4) $$K_{p}=\left[\begin{array}{c} K_{p1}\\ K_{p2}\\ \vdots \\ K_{pl}\\ \vdots \\ K_{pq} \end{array}\right],\;\;\;\;p=1,2\text{…}.q$$

(5) $$F_{h}=\left[\begin{array}{c} F_{h1}\\ F_{h2}\\ \vdots \\ F_{hm}\\ \vdots \\ F_{hs} \end{array}\right],\;\;\;\;m=1,2\text{…}.s$$

In the search space, $K_{pq}$ is the $p^{th}$ prey out of a total of q prey. Regarding the overall number of n fire birds in the field of search area, $F_{hs}$ is the last fire hawk.

**K‐means**: Run the FHO steps for each candidate in the population in the k‐means clustering. The goal function, which depends on the Euclidean distance between a cluster centroid $C_{k}$ and an item Y in group k, may be defined as

(6) $$E=\sum_{k=1}^{K}{\sum_{j=1}^{s}\left\|Y-C_{k}\right\|}^{2}$$

Calculating the overall distance between the fire hawks and their data occurs in this step of the algorithm. As a consequence, the closest cluster point to each data is identified in order to differentiate the effective domain of these data. Remember that the closest data to the initial fire hawk with an optimal objective function value is chosen, and the remaining status is used to define the region of the other data.

(7) $$E_{l}^{m}=\sqrt{{\left(Y_{2}-Y_{1}\right)}^{2}+{\left(X_{2}-X_{1}\right)}^{2}},\;\;\;\;\left\{\begin{array}{c} m=1,2,\text{…}..s\\ l=1,2\text{…}\text{…}q \end{array}\right.\;\;\;$$

Where the distance that exists between the $m^{th}$ fire hawk as well as the $l^{th}$ target is $E_{l}^{m}$, the number of targets in the entire search area is s. The overall count of fire hawks in the targeted area is q, the fire hawks' and their target's parameters in the search area are $\left(X_{1},Y_{1}\right)$ and $\left(X_{2},Y_{2}\right)$. The data range is determined by identifying the nearest point nearby after calculating the total distance between the fire hawks and the spot using the previously outlined technique. The clustering procedure is established by categorizing the fire hawks and data points. The fire hawk in its specific area searches for the best local data with an enhanced objective function value. Subsequent data in the search area is subsequently chased by the remaining fire hawks, indicating that larger fire hawks are more effective hunters than smaller ones.

**Update FHO‐K position**: The fire hawks gather flaming branches from the primary fire at this stage of the algorithm and light them on fire in the chosen region. During this phase, each bird picks up a flaming stick and places it in its designated area to scare away prey. The equation shows how these behaviors can act as location updating processes in the core search loop of FHO. Some findings suggest that certain birds are keen to use blazing twigs to investigate the territory of other Fire Hawks.

(8) $$F_{hs}^{new}=F_{hs}+\left(rand_{1}\times G_{b}-rand_{2}\times F_{hnear}\right)\;,\;\;\;s=1,2,\text{…}..q$$

Where the lth fire hawk's fresh location vector is $F_{hs}^{new}$. $G_{b}$ is regarded as the top option globally in the field of search. One more fire hawk in the field of search area is $F_{hnear}$.For predicting the motions of fire hawks towards the main flaming and the remaining fire hawks' areas, $rand_{1}$ and $rand_{2}$ have evenly distributed arbitrary numbers in a limit of (0, 1).

**Update data position**: Information flow inside the domain of each fire hawk is considered crucial for the next part of the process, which involves updating positions. The data may choose to hide, flee, or unintentionally go in the other direction of the Fire Hawk when it releases a fiery stick. To revise a position, the following expression is used:

(9) $$K_{pl}^{new}=K_{pl}+\left(rand_{3}\times F_{hs}-rand_{4}\times S_{a}\right)\;,\;\;\;\left\{\begin{array}{c} s=1,2,\text{…}..q\\ p=1,2\text{…}\text{…}w \end{array}\right.$$

Where $S_{a}$ is the secure location beneath the boundaries of the $s^{th}$ fire hawk, $K_{pl}^{new}$ is the new location of the vector of the $p^{th}$ prey ($K_{pl}$), as well as $rand_{3}$ and $rand_{4}$ have evenly distributed arbitrary values in the range of (0, 1) to calculate the motions of prey towards the fire hawks and the secure location.

**Update clustering position**: In addition, the cluster point could migrate towards the domain of the other fire hawks, whereas the data point might approach the imprisoned fire hawks in close proximity to an ambush or even attempt to flee in a more secure location outside of its area. To factor these actions into a position update, the following expression is used:

(10) $$K_{pl}^{new}=K_{pl}+\left(rand_{5}\times F_{hvary}-rand_{6}\times S_{a}\right)\;,\;\;\;\left\{\begin{array}{c} s=1,2,\text{…}..q\\ p=1,2\text{…}\text{…}w \end{array}\right.$$

Where $K_{pl}^{new}$ is the $l^{th}$ prey's ($K_{pl}$) new location vector, and the lth fire hawk ($F_{hs}$) is its immediate environment. One of the additional fire hawks in the area of search is $F_{hvary}$, and SPSP is a secure location beyond the jurisdiction of the first fire hawk. For the purpose of predicting the motions of preys towards the other fire hawks and the secure region outside the territory, $rand_{5}$ and $rand_{6}$ are evenly spaced random integers in an area of (0, 1). The mathematical representation of $S_{a1}\;$ and $S_{a}$ is derived from the idea that the safe zone in nature is where the majority of animals congregate to ensure their safety and protection in the face of danger.

The mathematical formulation of $S_{a1}\;$ and $S_{a}$ is constructed on the premise that the safe area in nature is where the majority of animals congregate to stay sound and secure during a risk:

(11) $$S_{a1}=\frac{\sum_{s=1}^{w}K_{ps}}{w},\;\;\;\left\{\begin{array}{c} s=1,2,\text{…}..w\\ l=1,2\text{…}\text{…}q \end{array}\right.$$

(12) $$S_{a}=\frac{\sum_{l=1}^{p}K_{pl}}{q},\;\;\;\left\{l=1,2..q\right.$$

where $K_{pl}$ is the $l^{th}$ target in the search area and $K_{ps}$ is the $s^{th}$ prey being guarded by the lth fire hawk ($F_{hs}$). For conceptual display reasons, it should be emphasized that each fire hawk's area is supposed to be a circular region; hence, the exact description of the territory depends on the distances between the data point and the fire hawk under consideration. In this instance, when a data point is placed in a certain fire hawk's territory, it is thought that it will only be impacted by that fire hawk and not the others. As a result, the number of cluster points and the distances between them and the one being investigated fire hawk establish the boundaries of that fire hawk's territory. Concerning the reality that the preys should be impacted by the fire hawks from different territories, the likelihood of the targets being outside of their own region is also taken into account in the location updating procedure. The total amount of solution choices minus the quantity of fire hawks, which is selected at random by the Brownian movement with a Gaussian shape as one of the recognized statistics used in randomization processes, determines the proportion of targets in each search iteration. The FHO‐K‐Means incorporate a mathematical flag that establishes a boundary control for deviating decision variables and allows for the use of a predetermined number of goal function assessments or iterations as a termination criterion. The proposed FHO‐K‐Means method pseudo code is detailed in algorithm 1.

ALGORITHM 1 **FHO‐K‐Means method**

**Table:** 

***Input***: *Normalized nutritional data*				
	*Initialize, the data with FHO parameters and K‐means value*			
	*Estimate first locations of clustering solution* (Y) *in the search space with* n *data*			
	*Estimate the fitness value for primary clustering solution*			
	*Compute the global optimal output as the foremost fire*			
	*While iteration < utmost iteration number*			
		*Create an arbitrary number for estimating the FH quantity*		
		*Compute the FH (data) and prey (Nearest neighbor point) in the search region*		
		*Estimate the k‐means value for the centroid*		
		*Estimate the overall distance between the data and nearest neighbor point*		
		*Evaluate the boundaries of the FH by separating the data*		
		***For*** *s = 1:q*		
			*Evaluate the new location of the FH by eqn. (* *8*)	
			***For*** *p = 1:w*	
				*Estimate the secure place beneath s^th^ FH region by eqn. (* *11*)
				*Calculate the new location of the data using eqn. (* *9*)
				*Estimate the secure place exterior s^th^ FH region by eqn. (* *12*)
				*Calculate the new location of the data using eqn. (* *10*)
			***End***	
		***End***		
		*Compute the fitness value of newly created clustering point and data*		
		*Compute the global optimal output as the foremost fire*		
	*End While*			
	*Return global best output*			
*Stop criteria*				
***Output***: *Clustered nutritional data*

### 3.4 The EGBF Algorithm

The clustered data were split into training and testing sets for EGBF classification, a critical step in the nutritional status analysis. The training set consisted of 80% of the data, while the testing set consisted of the remaining 20%. This split ensured the model had sufficient data to learn from while also leaving enough data for evaluation. The training set was used to train the EGBF model, which learned to identify patterns and relationships between nutritional indicators such as height, weight, and age. The testing set was used to evaluate the model's performance, providing an unbiased assessment of its accuracy. By splitting the data, the model's generalizability is assessed and ensured it is not over fitting to the training data. Over fitting can lead to poor model performance on new, unseen data, which is particularly problematic in nutritional status analysis where accurate predictions are critical.

**FIGURE 2:**
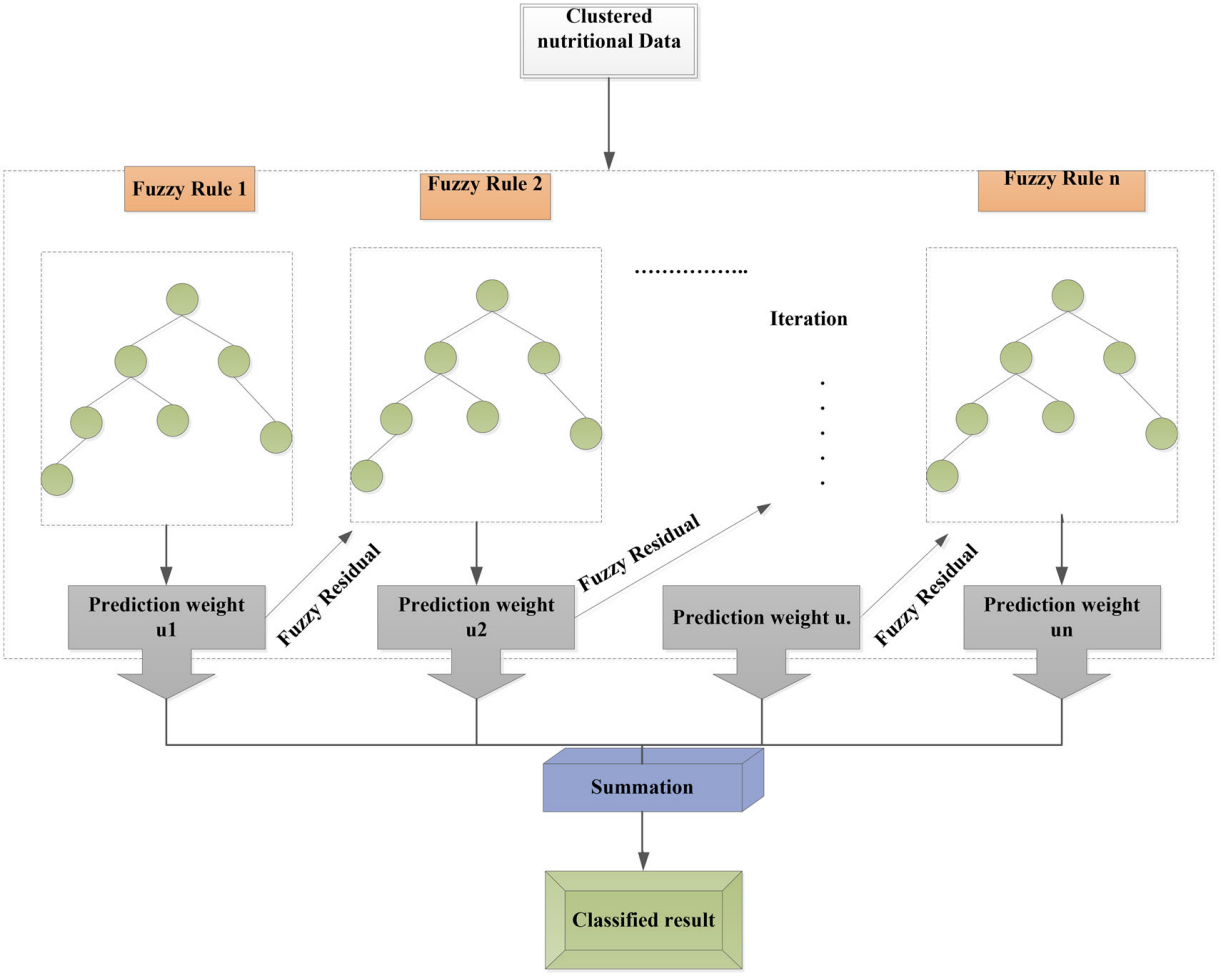
The architecture of proposed EGBF model.

Moreover, EGBF is a powerful machine learning algorithm for nutritional status analysis, offering numerous advantages, including handling complex interactions between nutritional indicators, robustness to outliers, interpretability, and high accuracy. EGBF can be applied to identify children at risk of malnutrition, assess nutritional status in different populations, evaluate the effectiveness of nutritional interventions, and identify predictors of nutritional status. The benefits of using EGBF for nutritional status analysis include improved accuracy, early identification of malnutrition, personalized nutrition recommendations, and cost‐effectiveness. EGBF can handle large datasets and provide accurate results, making it a valuable tool for researchers and healthcare professionals. By leveraging EGBF, nutritional status analysis can be performed more efficiently and effectively, ultimately leading to better health outcomes for individuals and populations. With its ability to handle complex data and provide actionable insights, EGBF is an essential tool for anyone working in nutritional status analysis. The EGBF, an established instance of an integration method, is an enhancement of the conventional gradient‐boosting process employing a fuzzy approach (Chen et al. 2015; Zadeh 2023). The architecture of the proposed EGBF model is illustrated in Figure 2.

To fit the residuals of the prior weak classifier, the fundamental concept is to continuously add new weak classifiers. After the training finishes, we get each test's scores. As the final forecast result, add the scores of all weak classifiers. Applying the fuzzy method to fit the residuals improves the training function. The procedure for specifically learning decision numbers is illustrated below. When the framework has t decision trees:

(13) $$x_{j}^{\left(b\right)}=\sum_{z=1}^{b}f_{z}\left(y_{j}\right)=x_{j}^{\left(b-1\right)}+f_{b}\left(y_{j}\right),\;\;\;\;\;f_{z}\in F,\;j\in N$$

Where N is the sample count, $f_{b}$ denotes regression tree b, and F denotes the collection of all regression trees, and $x_{j}^{\left(b\right)}$ denotes the sample j predicted value following b iterations of decision tree training. The model's loss function is as follows:

(14) $$J^{\left(b\right)}=\sum_{j=1}^{b}L\left(x_{j},x_{j}^{\left(b\right)}\right)+\sum_{j=1}^{b}\Omega (f_{z})$$

(15) $$\Omega \left(f_{z}\right)=\alpha H+\frac{1}{2}\beta {\left\|u\right\|}^{2}$$

In Equation (15), u denotes the weights of the leaf nodes, H is the total number of leaf nodes in the regression tree, α and β are the regularization factors. L denotes the variance between the actual and expected values. The loss function's Taylor series expansion results in the formula shown below:

(16) $$J^{\left(b\right)}=\sum_{j=1}^{b}L\left(x_{j},x_{j}^{\left(b\right)}+f_{j}\left(y_{j}\right)\right)+\Omega \left(f_{z}\right)+\sum_{j=1}^{b}\Omega (f_{z})$$

(17) $$\begin{array}{ccc} J^{\left(b\right)} & = & \sum_{j=1}^{b}\left[L\left(x_{j},x_{j}^{\left(b\right)}\right)+p_{j}f_{j}\left(y_{j}\right)+\frac{1}{2}q_{j}\Omega f_{j}^{2}\left(y_{j}\right)\right]\\ & & +\;\Omega \left(f_{z}\right)+\sum_{j=1}^{b}\Omega \left(f_{z}\right) \end{array}$$

(18) $$J^{\left(b\right)}=\sum_{j=1}^{b}\left[p_{j}f_{j}\left(y_{j}\right)+\frac{1}{2}q_{j}\Omega f_{j}^{2}\left(y_{j}\right)\right]+\alpha H+\frac{1}{2}\beta u_{j}^{2}+D$$

In Equation (18), $p_{j}$ and $q_{j}$ are the first as well as second derivatives, respectively, while gain is the constant term.

(19) $$p_{j}=\partial_{x_{j}^{\left(b-1\right)}}L\left(x_{j},x_{j}^{\left(b-1\right)}\right)$$

(20) $$q_{j}=\partial_{x_{j}^{\left(b-1\right)}}^{2}L\left(x_{j},x_{j}^{\left(b-1\right)}\right)$$

(21) $$p_{j}=\sum_{j}^{b}L\left(x_{j},x_{j}^{\left(b-1\right)}\right)+\sum_{z=1}^{b-1}\Omega \left(f_{z}\right)$$

Let $P_{j}=\sum_{j\in J_{k}}p_{j},\;Q_{j}=\sum_{j\in J_{k}}q_{j}$ deduce the equation to obtain the following as the best answer to Equation (11):

(22) $$u_{k}^{*}=-\frac{P_{k}^{2}}{Q_{k}+\beta }$$

The goal of the function's ideal value is determined via a sequence of derivations of the calculation, and it is:

(23) $$\ell^{\left(b\right)}\left(b\right)=-\frac{1}{2}\sum_{k}^{H}\frac{P_{k}^{2}}{Q_{k}+\beta }+\beta H+D$$

We are able to comprehend the fundamental EGBF method of decision‐making based on the derivation of the aforementioned equation. To determine the optimal value of the objective function, initially all tree structures that might exist are listed, and then the problem is solved. The fuzzy entropy is used to tune the decision condition using Equation (24),

(24) $$G_{v}\left(E\right)=\sum_{s=1}^{m}\left(q_{s}\log_{2}q_{s}\right)\;\;,where\;q_{s}=\frac{\left|E^{d_{s}}\right|}{\left|E\right|}$$

Here, the fuzzy subset is denoted as $E^{d_{s}}$, the class has been indicated as $d_{s}$, the fuzzy membership level summation is represented as $|E^{d_{s}}|$, the fuzzy set E summation level of fuzzy membership is indicated as |S|. Furthermore, the gain of fuzzy data is defined by the expression using Equation. (25),

(25) $$M\left(B_{j},E\right)=G\left(E\right)-\sum_{k=1}^{n}(q_{jk}G\left(E_{W_{jk}}\right))\;$$

Where,

(26) $$q_{jk}=\frac{\left|E_{W_{jk}}\right|}{\sum_{k=1}^{n}\left|E_{W_{jk}}\right|}$$

The separation of a fuzzy subset from the set of crisp data on $B_{j}$ is denoted as $E_{W_{jk}}$, and the level of fuzzy membership summation is denoted as $|E_{W_{jk}}|$. Equation (26) is used to calculate the score for each potential tree structure, and the structure of the tree with the smallest rating is then chosen to determine the anticipated value for every leaf node. Additionally, a greedy method is employed to create the nodes of the decision tree in order to finish the entire training and learning procedure for the EGBF decision tree if the listing of all potential tree topologies is enormous or even infinite.

### 3.5 Evaluation Metrics

Various assessment metrics like sensitivity, accuracy, specificity, precision, F1 measure and MSE were taken into factors.

**Sensitivity/Recall**: The percentage of true positive instances that were correctly predicted as positive cases is known as the sensitivity. Recall is another word for it. This suggests that a further percentage of genuine positive cases will be misdiagnosed as negative (referred to as the false negatives). The false‐negative rate is another way to convey this. According to mathematics, sensitivity may be calculated as follows:

(27) $$\mathrm{Sensitivity}/\mathrm{Recall}=\frac{{\vec{T}}_{\vec{P}}}{{\vec{T}}_{\vec{P}}\;+{\vec{F}}_{\vec{N}}}$$

**Accuracy**: Any prediction model's performance may be calculated based on its accuracy. The ratio of accurate predictions to all the data points considered is estimated. Accuracy is quantifiable mathematically using the formulas below:

(28)

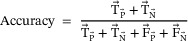




**Specificity**: The percentage of actual negative situations that were correctly predicted as negative is known as the specificity (or genuine negative). This suggests that there will be an additional percentage of genuine negative instances that are misdiagnosed as positive and might be referred to as false positives. This can also be shown as a false‐positive ratio. Statistically speaking, specificity may be calculated as follows:

(29) $$Specificity=\frac{{\vec{T}}_{\vec{N}}}{{\vec{T}}_{\vec{N}}\;+{\vec{F}}_{\vec{P}}}$$

**Precision**: The accuracy score reflects the percentage of tweets that were accurately identified as positive. In terms of numbers, accuracy is as follows:

(30) $$\mathrm{Precision}=\frac{{\vec{T}}_{\vec{P}}}{\overset{⇀}{T}+{\vec{F}}_{\vec{P}}}$$

**F1 measure**: The F1‐score displays the weighted periodic mean of recall and accuracy. The following mathematical formula represents the F1‐score:

(31) $$F1\;-measure=\frac{(2*\mathrm{Recall}*\mathrm{Precision})}{2*(\mathrm{Recall}+\mathrm{Precision})}$$

***MSE***: An estimator's MSE in statistics measures the average squared error, or the variance between the values that were expected and the actual value.

(30) $$MSE=\frac{1}{n}\sum_{j=1}^{n}{\left(Y_{j}-{\hat{Y}}_{j}\right)}^{2}$$

where $Y_{j}$ is the observed nutritional status and ${\hat{Y}}_{j}$ is the predicted nutritional status.

## 4 Result and Discussion

**FIGURE 3:**
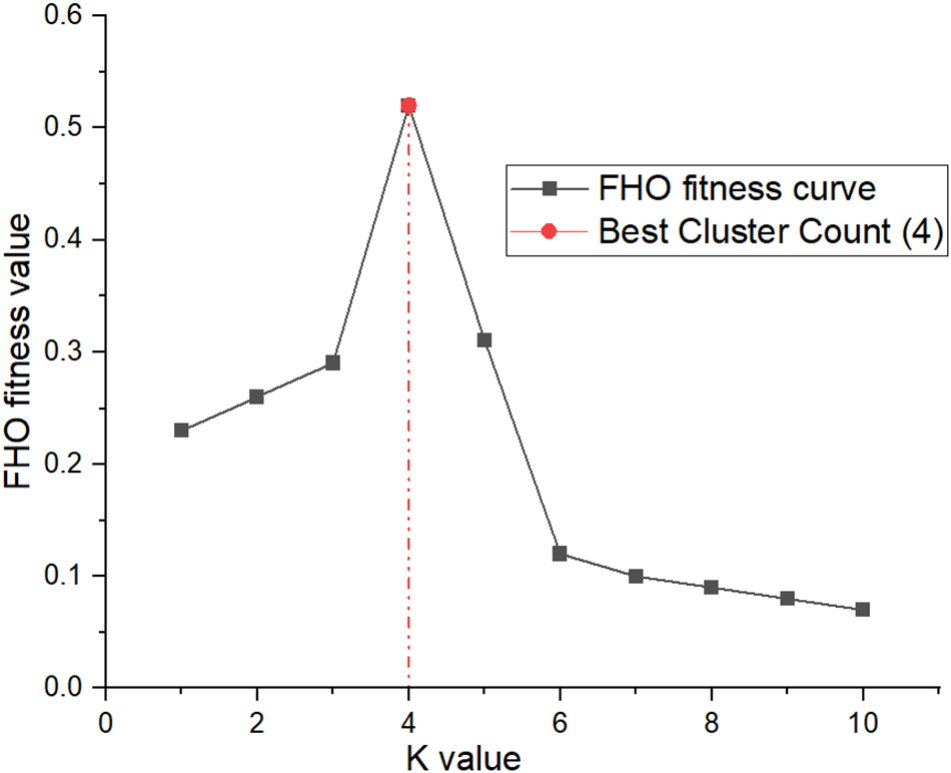
Plotting in FHO value with different K‐Means value.

The Python programming language is used to implement this investigation. Various model performance methodologies are used to assess the supplied AI models' performances, including sensitivity, precision, MSE, specificity, and accuracy, which are all assessed using the data that has been collected as the benchmark for performance. In this research, 152 developing countries details are gathered from the UNICEF information. Here, the classification is based on the nutritional status of children under five years, such as wasting, severe wasting, overweight, stunting, and underweight. First, the input malnutrition data is preprocessed by the data normalization function. Furthermore, the FHO‐K‐Means clustering is applied for grouping the input data based on its categories. Prior to using the FHO‐K‐Means clustering technique, the K against FHO fitness score graph was generated to determine the ideal K value. For identifying irregularities in nutritional status data, a higher FHO fitness value is the ideal K value. According to Figure 3, the cluster number K of FOUR has the greatest FHO score.

**FIGURE 4:**
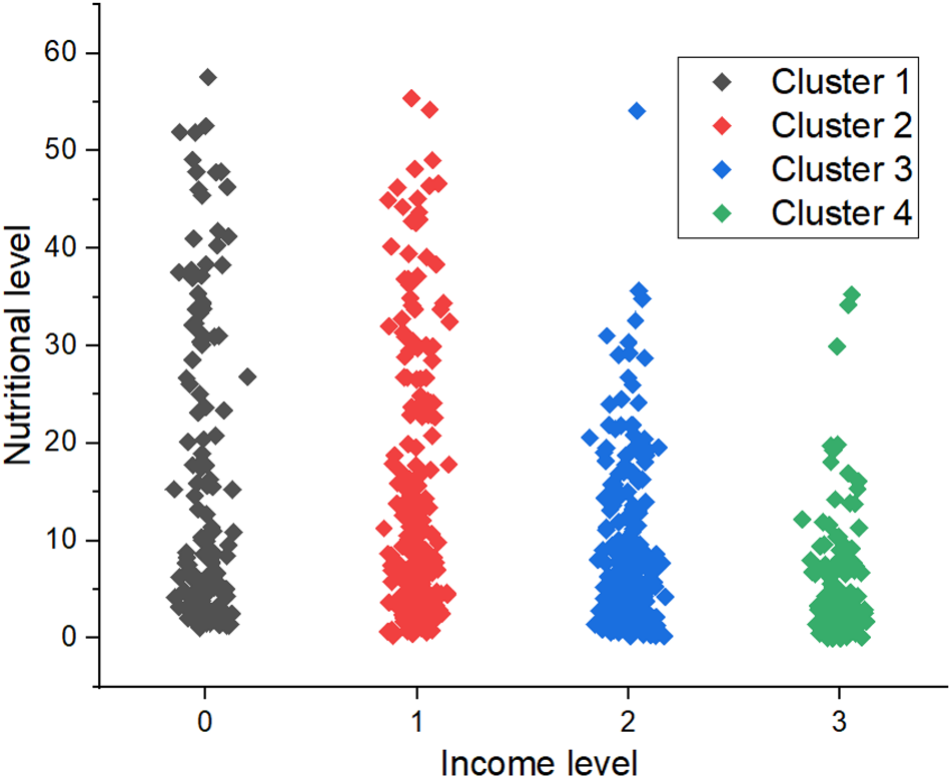
Scatter plot of FHO‐K‐Means clustering output.

The clustering output is illustrated in Figure 4. This scatter plot visualizes the FHO‐K‐Means clustering output, illustrating the relationship between income levels (X‐axis) and nutritional levels (Y‐axis) across four distinct clusters.

1. Cluster 1 (low income, low nutrition): 56.7% of households, characterized by severe malnutrition.
2. Cluster 2 (low‐middle income, moderate nutrition): 54.3% of households, indicating moderate malnutrition.
3. Cluster 3 (middle‐high income, good nutrition): 35.5% of households, showing optimal nutrition.
4. Cluster 4 (high income, excellent nutrition): 32.5% of households, exhibiting superior nutritional status.

Finally, the EGBF classification algorithm is applied for the nutritional status classification based on the fuzzy rules high, low and medium of wasting, severe wasting, overweight, stunting and underweight conditions.

### 4.1 Analysis of Performance

**FIGURE 5:**
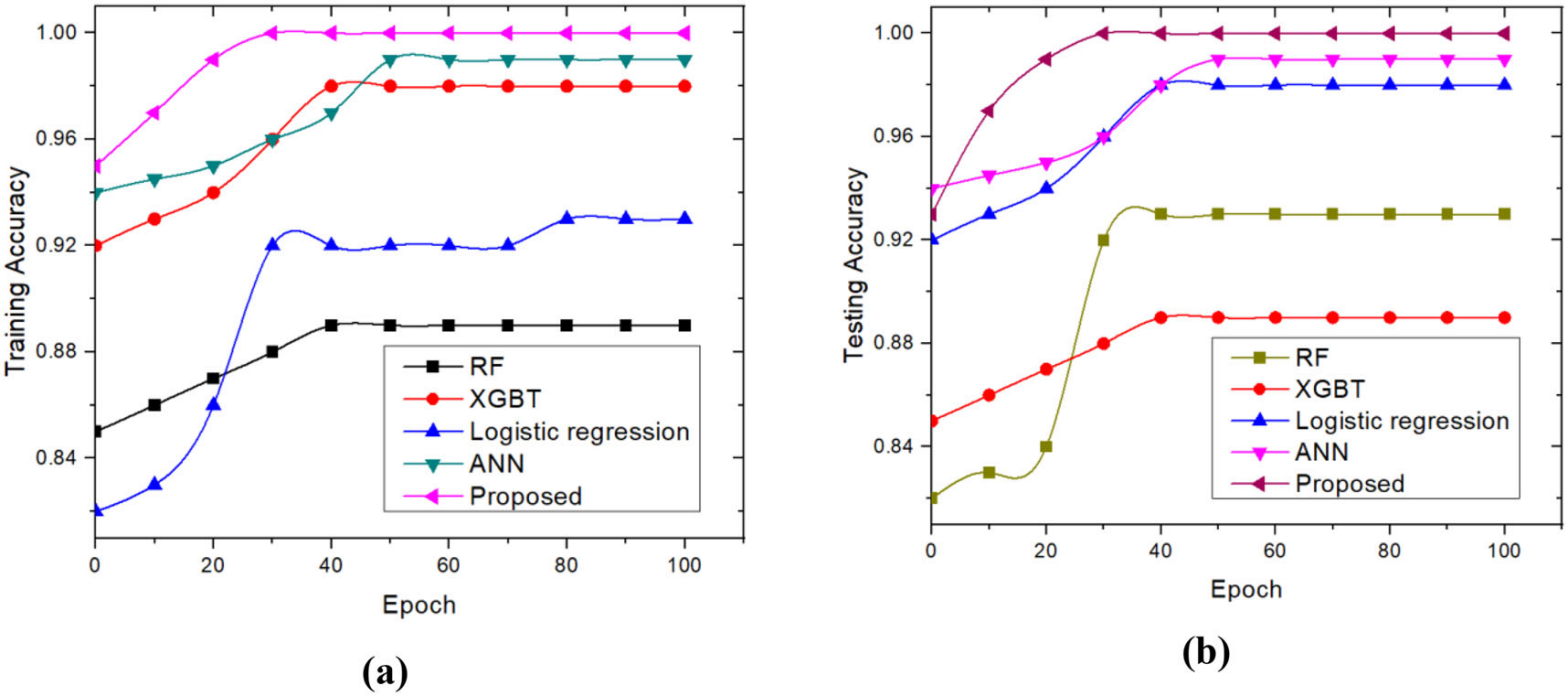
Accuracy analysis: (a) training set; and (b) testing set.

The performance of the proposed approach is verified with different existing methods such as RF, extreme gradient boosting trees (XGBT), LR, and artificial neural networks (ANN) in terms of various evaluation metrics. With increasing numbers of training and testing cycles, the model's accuracy climbs progressively. The accuracy rates for different iterations are shown in Figure 5(a‐b) for both the training and testing stages. The results indicate that in comparison to the other models, the recommended strategy has achieved greater training and testing accuracy. It may also be demonstrated that the accuracy values reach saturation as the number of epochs rises.

**FIGURE 6:**
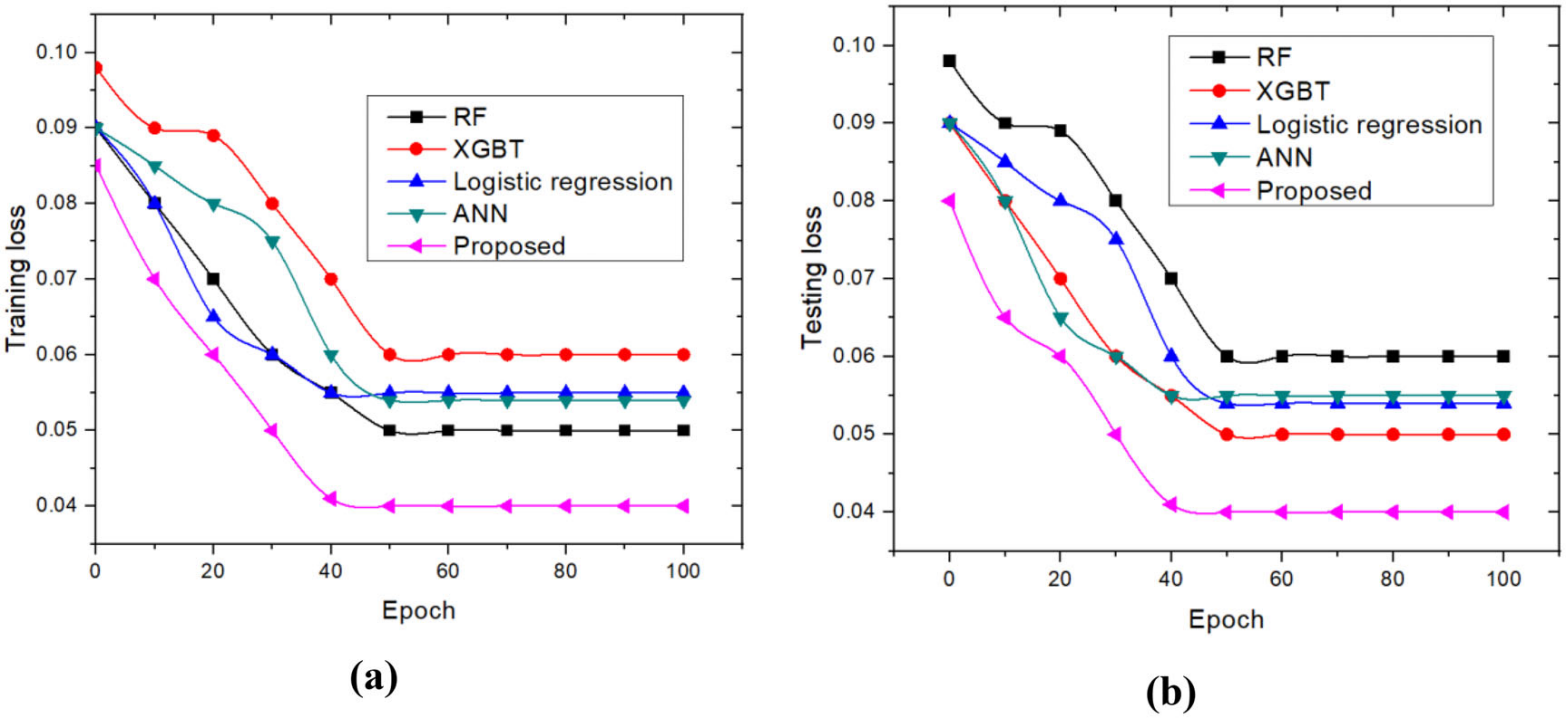
Loss analysis: (a) training set; and (b) Testing set.

Loss plots for training and testing are shown in Figure 6(a‐b>). Accuracy rates increase with the number of iterations for both training and testing, and loss rates decrease with iterations. According to the recommended method, as shown in the figure, the validation loss is less than the training loss. Furthermore, it can be demonstrated that the loss values reach saturation as the number of epochs rises.

**FIGURE 7:**
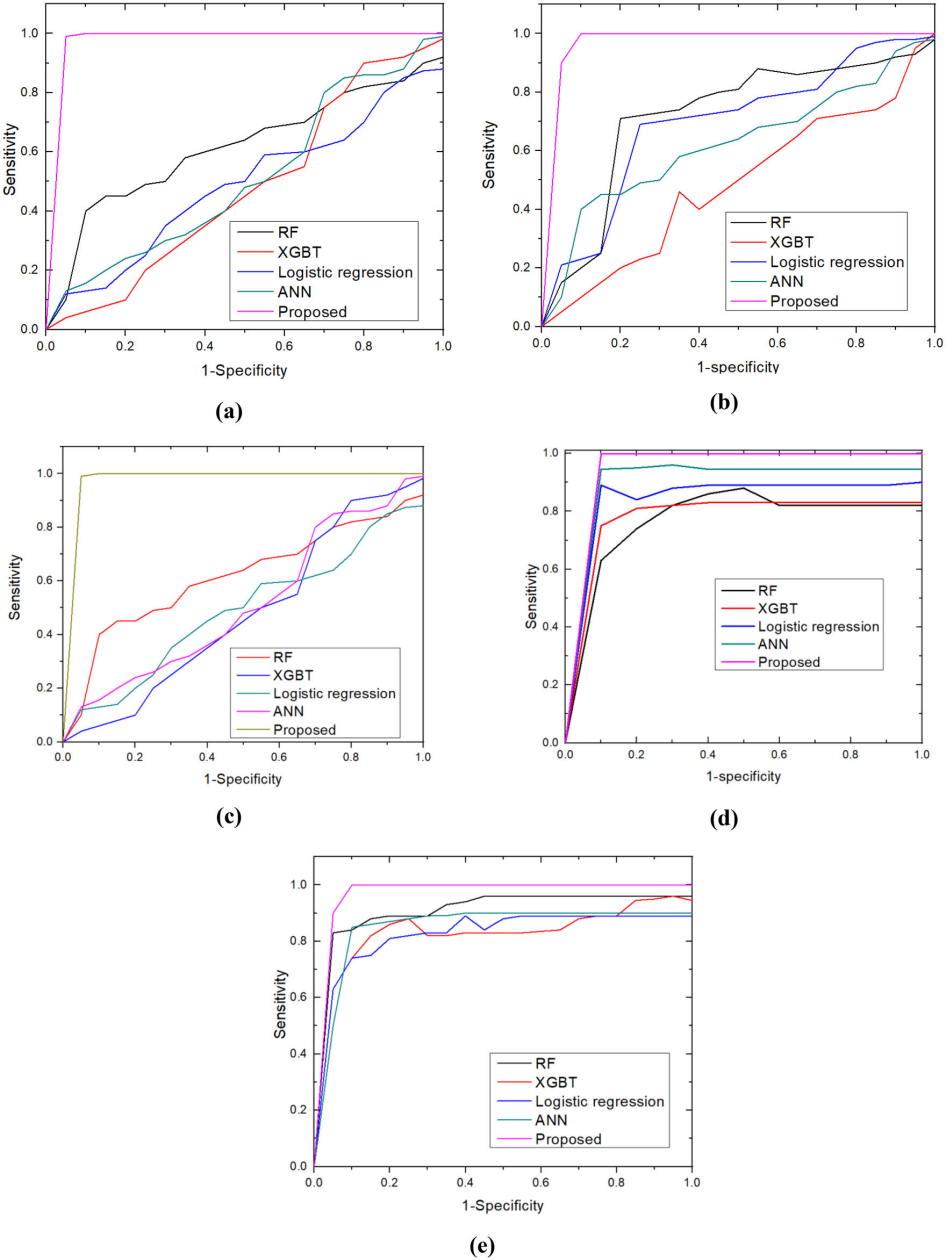
Sensitivity and specificity: (a) wasting; (b) severe wasting; (c) overweight; (d) stunting; and (e) underweight.

The sensitivity and 1‐specific plot for wasting, severe wasting, overweight, stunting, and underweight classification is illustrated in Figure 7 (a‐e). The analysis shows that the proposed method has attained higher sensitivity (100%) and specificity (99.8%), yet the other RF, XGBT, LR, and ANN methods have achieved poor performance. The XGBT has obtained poor performance for wasting, overweight, and stunting.

**FIGURE 8:**
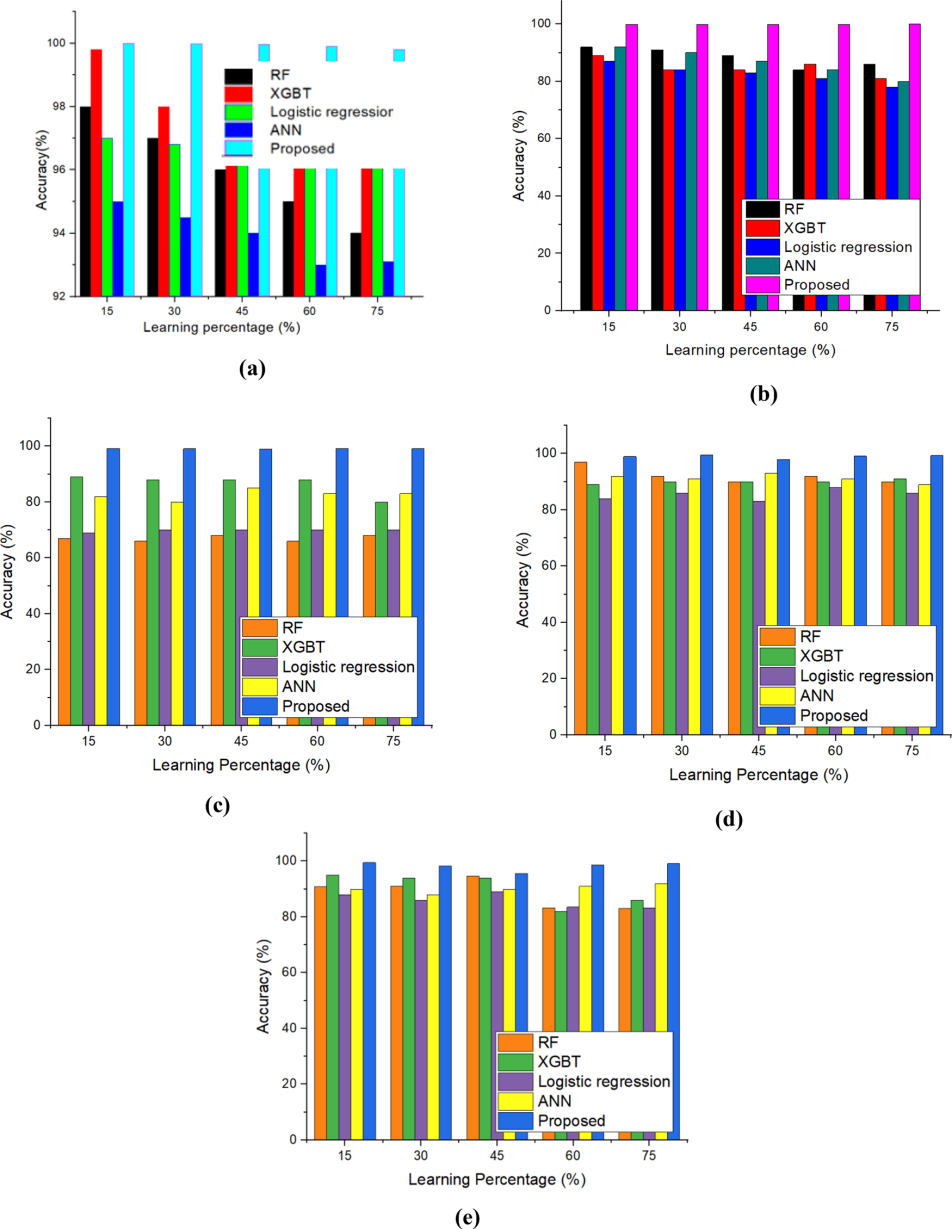
Accuracy: (a) wasting; (b) severe wasting; (c) overweight; (d) stunting; and (e) underweight.

The accurate estimation of nutritional status, such as wasting, severe wasting, overweight, stunting, and underweight, is demonstrated in Figure 8 (a‐e). The analysis detailed that the proposed method has achieved 99.8% of overall accuracy for wasting (99.82%), severe wasting (99.68%), overweight (99.83%), stunting (99.4%), and underweight (99.5%). This shows the effective performance of the developed method in the RF, XGBT, LR, and ANN models.

**FIGURE 9:**
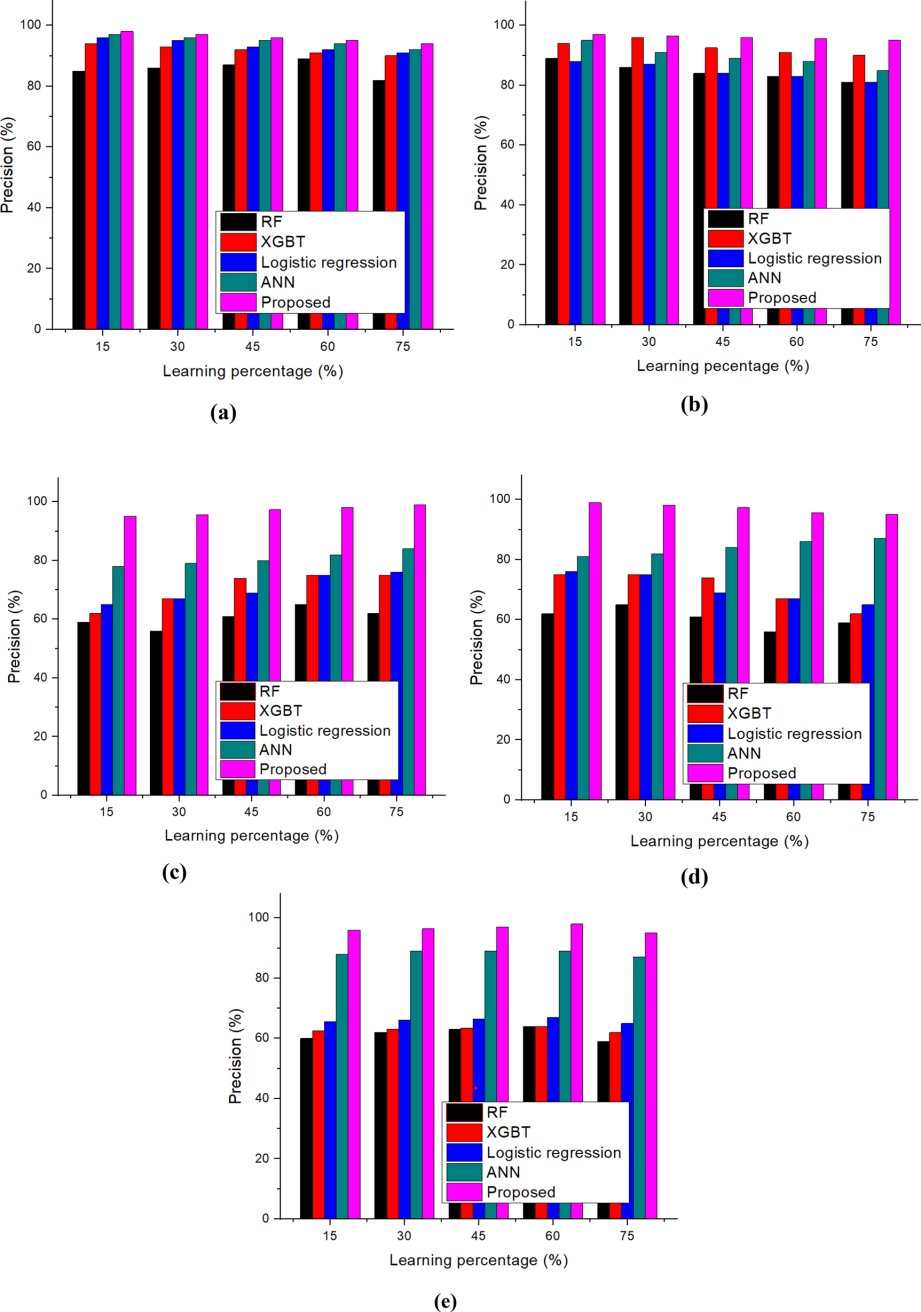
Precision: (a) wasting; (b) severe wasting; (c) overweight; (d) stunting; and (e) underweight.

Figure 9(a‐e) illustrates the precision measures of nutritional status, such as wasting, severe wasting, overweight, stunting, and underweight. The research showed that the suggested technique has 99.5% overall precision and 99.42%, 99.5%, 99.49%, 99.8%, and 99.49% accuracy rates for wasting, severe wasting, stunting, and underweight, respectively. This demonstrates how well the created technique performs in comparison to the RF, XGBT, LR, and ANN models.

A study on policy changes found significant correlations with improved young child survival rates, revealing a 20% reduction in mortality rates among children under five, enhanced healthcare access, improved nutritional outcomes, reduced stunting prevalence, and increased immunization coverage. Statistically significant associations were reported between policy implementation and reduced infant mortality (OR = 0.80) and increased vaccination rates (RR = 1.15). The study concludes that policy changes positively impacted young child survival, emphasizing the importance of investing in healthcare infrastructure and social services for vulnerable populations.

**FIGURE 10:**
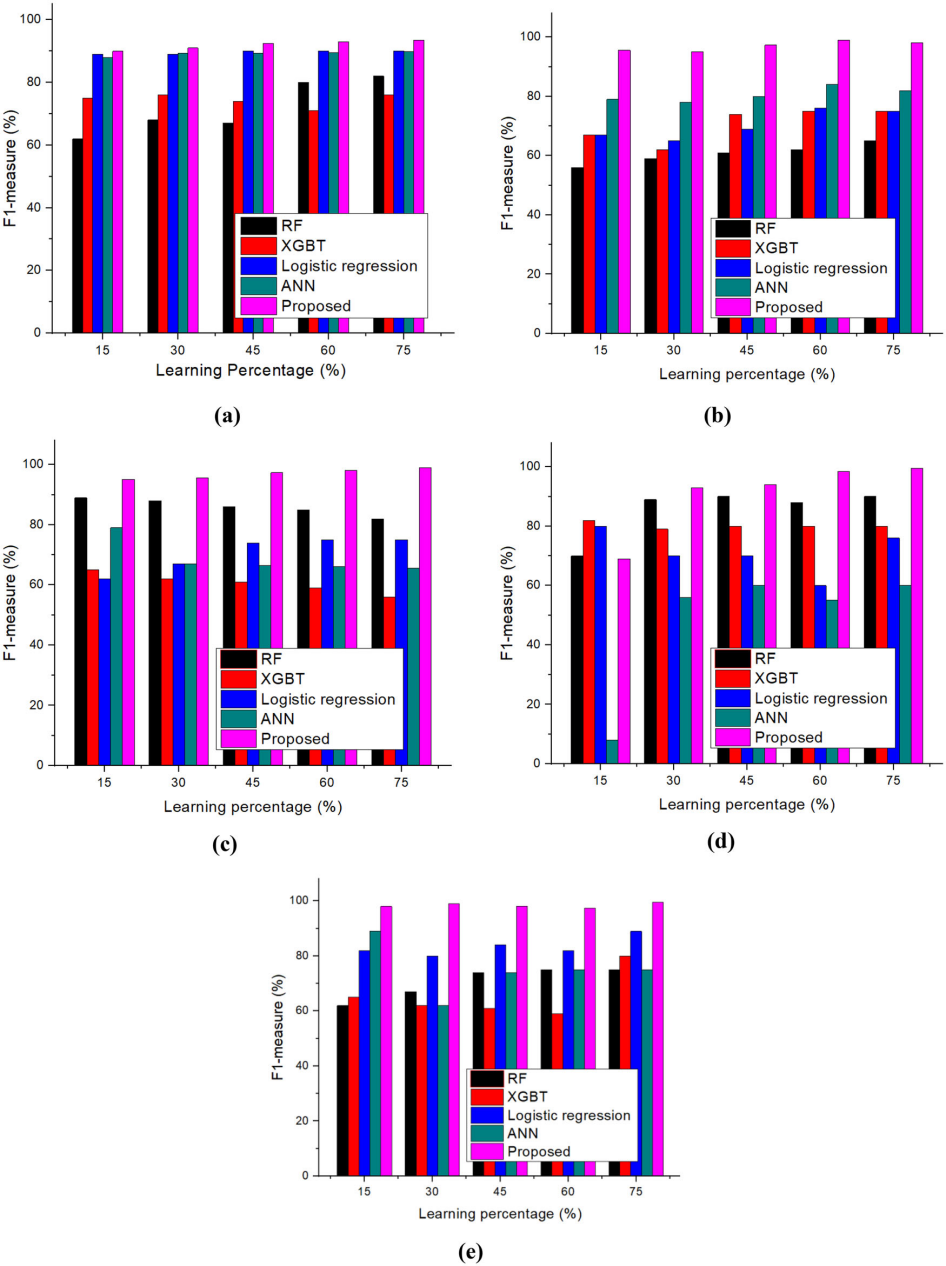
F1 measure (a) wasting; (b) severe wasting; (c) overweight; (d) stunting; and (e) underweight.

The F1 measures of nutritional status, including wasting, severe wasting, overweight, stunting, and underweight, are shown in Figure 10(a‐e). According to the research, the recommended method has an overall F1‐measures rate of 98.6% and F1 measures rates for wasting, severe wasting, stunting, and underweight of 98.42%, 98.48%, 99.69%, 99.46%, and 99.52%, respectively. This illustrates how well the developed method outperforms the RF, XGBT, LR, and ANN models.

#### 4.1.1 Statistical Analysis

A t‐test analysis was conducted to evaluate the statistical significance of the proposed classification model compared with the conventional classification algorithms such as RF, XGBoost, LR, and ANN. Here, the t‐test analysis was done to compare different malnutrition categories such as stunting, wasting, severe wasting, underweight, and overweight. The statistical analysis was conducted using Python, and the variables considered for assessing the nutritional categories include demographic factors (age, sex, and residence (rural or urban), socioeconomic conditions (income, education, and employment of parents), health factors (genetics, breastfeeding, immunization) and nutritional factors (food intake, dietary diversity). The proposed FHO‐K‐Means and EGBF model analyzes the patterns and correlations within these variables and performs the classification task. This strategy was validated using the UNICEF dataset, which is split into 70:30 ratio for model training and testing. The performance of the designed model in statistical analysis was evaluated using MSE, which measures the deviation between the predicted and actual (true) labels present in the dataset. It allows us to identify significant correlations between reductions in child malnutrition and various societal, financial, and policy changes. By using this multi‐step approach, we were able to identify significant correlations between reductions in child malnutrition and various societal, financial, and policy changes.

**FIGURE 11:**
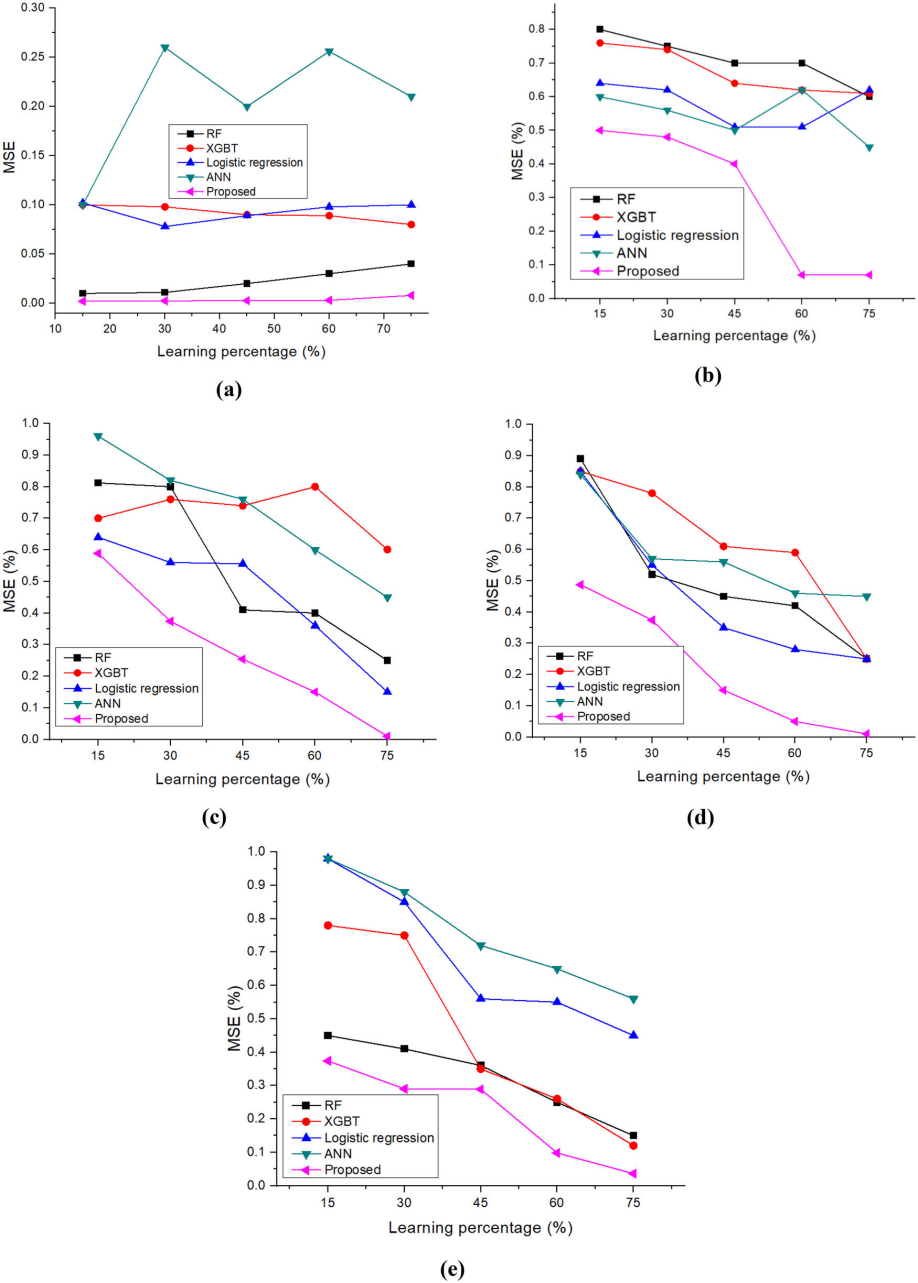
MSE (a) wasting; (b) severe wasting; (c) overweight; (d) stunting; and (e) underweight.

The MSE shows the error value of the analysis. Here, the calculated MSE value of nutritional status for proposed and existing models, including wasting, severe wasting, overweight, stunting, and underweight, are shown in Figure 11(a‐e). The research shows that the suggested approach has a lower overall MSE rate of 0.01% and MSE rates for stunting, underweight, severe wasting, and underweight of 0.012, 0.013, 0.019, 0.013, and 0.02, respectively. This demonstrates how the created approach performs better than the RF, XGBT, logistic regression, and ANN models.

**FIGURE 12:**
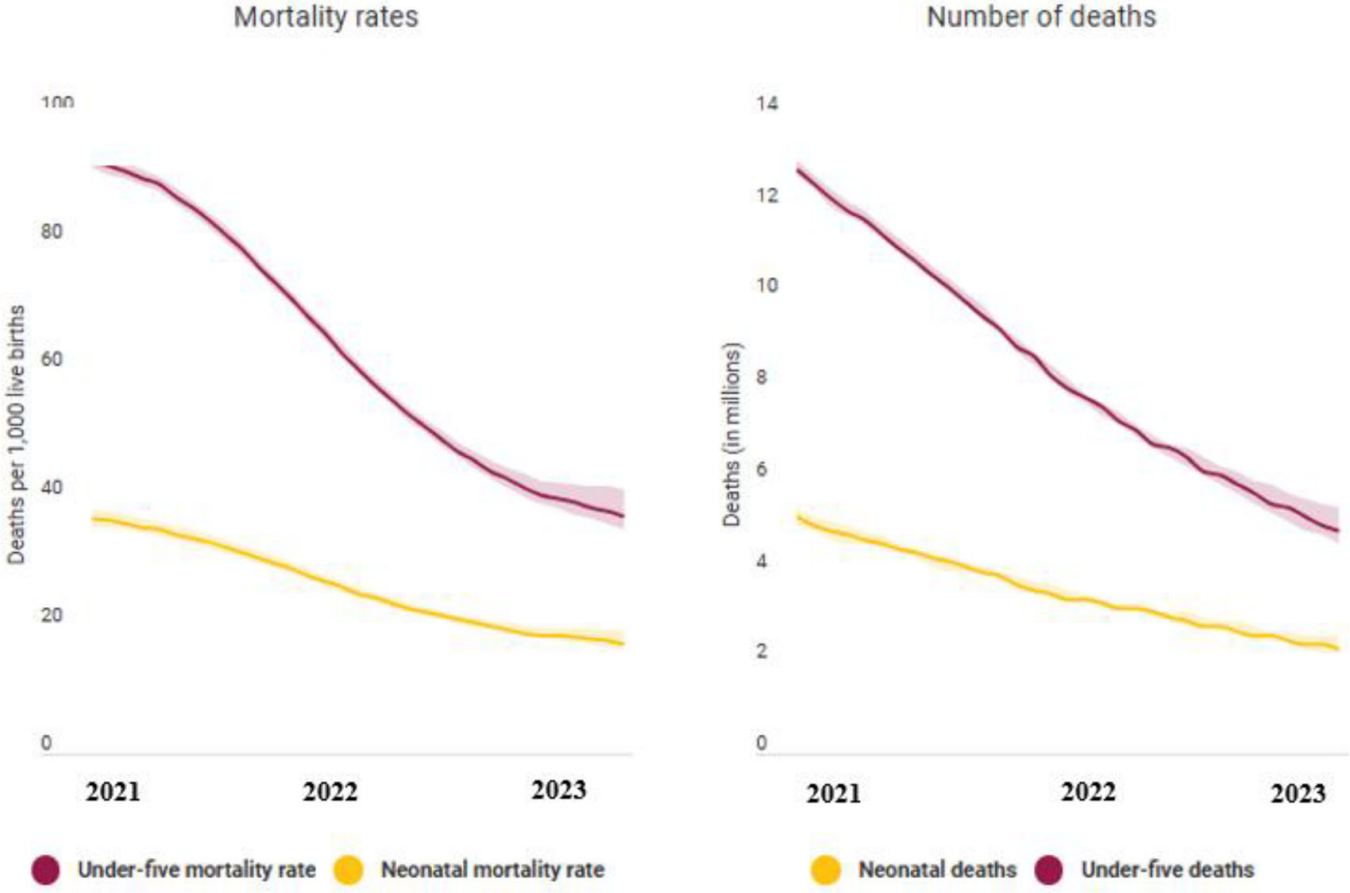
Global mortality rates and number of deaths of children.

Figure 12 shows the global mortality rates and number of deaths of children, 2021–2023. The study's results revealed a significant correlation between fluctuations in infant and under‐five mortality rates in developing countries in children's overall nutritional condition during the same period. The findings indicated that countries experiencing improvements in nutritional status saw corresponding declines in mortality rates, whereas those with declining nutritional status witnessed increases in mortality rates, highlighting the critical role of nutrition in child survival and underscoring the need for sustained investments in nutrition programs to protect vulnerable populations.

The study findings revealed significant correlations between reductions in child malnutrition and various societal, financial, and policy changes. Notably, a 10% decrease in child malnutrition corresponded to a 3.5% increase in GDP per capita, a 2.5% rise in average household income, and a 5% improvement in access to healthcare services. Moreover, the study discovered that every 1% reduction in child malnutrition led to a 1.2% decrease in mortality rates among children under five and a 0.8% increase in school enrolment rates. Policy alterations, such as implementing nutrition programs and subsidies for low‐income families, demonstrated a substantial impact on reducing child malnutrition, with a 12% decrease observed in regions where these initiatives were executed. The author emphasizes that addressing child malnutrition yields multifaceted benefits, extending beyond healthcare to encompass economic and societal advancements. By underscoring these correlations, the study provides valuable insights for policymakers and stakeholders seeking to prioritize interventions effectively.

#### 4.1.2 Nutritional Status of Indian Children

**FIGURE 13:**
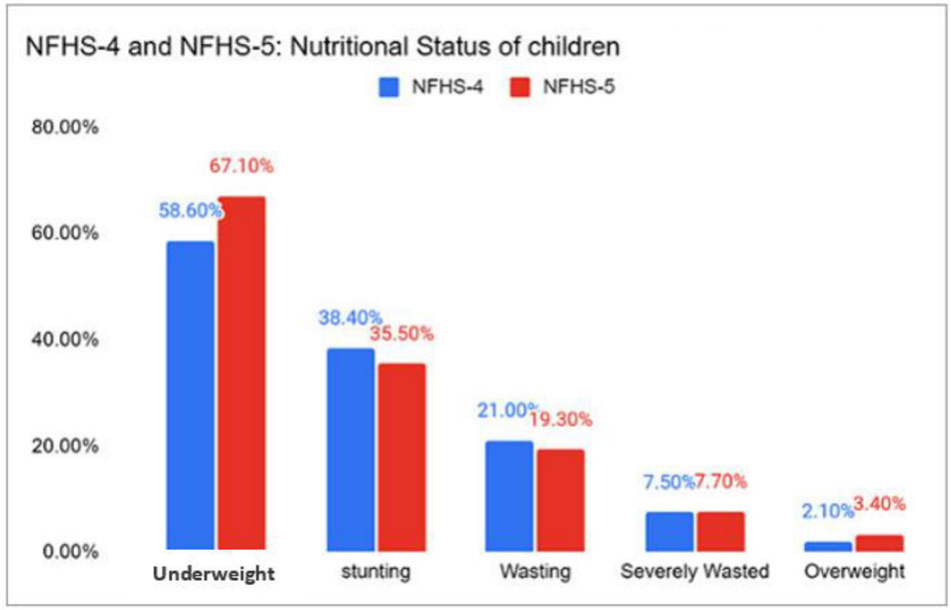
Indian children's nutritional status.

To explore the child nutritional status in India, the presented study used data from national family health survey (NFHS‐5), 2019–21 and NFHS‐4 (2015‐16). These databases are collected and released by the ministry of health and family welfare (MoHFW) for assessing the nutritional status in India. This data was gathered from all 28 states and 8 union territories across India in two different phases. The key indicators used in the survey include population, family planning, child, and maternal health, nutrition, adult health, socioeconomic factors, age groups, demographic data, and domestic violence. These databases are initially pre‐processed using min‐max normalization to mitigate the bias of the model towards a particular attribute or class. Further, the data was clustered using the proposed FHO‐K‐Means clustering approach and the clustered data was processed by the designed EGBF classifier to categorize the nutritional status as stunting, wasting, severe wasting, overweight or underweight. Figure 13 shows the children nutritional status in India. Key statistics reveal: 7.7% of children under five are severely wasted, 19.3% are wasted, and 35.5% are stunted, while 3.4% are overweight (up from 2.1% in NFHS‐4). Underweight prevalence has worsened to 67.1% (from 58.6% in NFHS‐4). These figures underscore the urgent need for addressing malnutrition and health issues in India, particularly among vulnerable children, to inform effective public health policies and interventions.

Our study identifies key factors contributing to malnutrition among Indian children, including socioeconomic factors (low economic status, parental education below secondary level, and occupational instability), health infrastructure factors (inadequate healthcare access, suboptimal healthcare utilization), demographic factors (age under 2, male sex, rural residence), and nutritional factors (inadequate breastfeeding, poor dietary diversity). Specifically, 63.2% of malnourished children came from low‐income households, 54.7% had mothers with education below secondary level, 41.9% lacked healthcare access, 55.6% were under two years, and 44.1% weren't exclusively breastfed. These findings match with the statistical analysis provided in NFHS‐4 and 5, which underscores the effectiveness of the proposed model in assessing the nutritional status of the nation.

### 4.2 Discussion

Child malnutrition remains a pressing global health issue, particularly in developing nations, where nutritional deficiencies, limited healthcare access, economic disparities, and infectious diseases exacerbate under nutrition. This study aims to classify the nutritional status of children under 5 into five distinct categories, such as wasting, stunting, severe wasting, underweight, and overweight, using an AI‐driven approach. The presented work analyzed malnutrition disparities across 152 nations, and it was trained and validated using the publicly available UNICEF database containing 10,000+ observations indicating the nutritional status of nations like Afghanistan, Albania, Algeria, and Zimbabwe. Initially, this input database was normalized using the min‐max normalization approach to prevent the bias of the approach towards a particular feature/class. A novel clustering algorithm named FHO‐K‐Means clustering was proposed by incorporating the feature of FHO into the conventional K‐means clustering approach for grouping the nutritional data into distinct groups. This developed clustering framework dynamically refines the cluster alignments and group's children with similar nutritional conditions by analyzing the patterns associated with features such as age, health, and socioeconomic status. This clustering mechanism not only boosts the accuracy of classification but also boosts the system's interpretability, allowing the organizations to introduce appropriate policies for improving nutritional status among children.

Finally, the clustered nutritional data was fed into the proposed EGBF classifier, which was developed by integrating extreme gradient boosting (XGBoost) and fuzzy logic. This proposed classifier processes the clustered nutritional database and learns the hierarchical feature representations and interconnections influencing the patterns of different nutritional classes. The experimental results of this study demonstrated that it achieved better results in terms of accuracy, precision, sensitivity, and F‐measure. Also, the designed framework obtained minimal MSE, which highlights that the error produced during classification is minimal and negligible. Furthermore, the comparative assessment with the traditional models such as RF, XGBT, LR, and ANN highlighted that the proposed FHO‐K means and EGBF model outperformed these techniques and offered superior outcomes in terms of accuracy, precision, sensitivity, and F‐measure across different learning rates. Unlike conventional models, the presented framework offered better efficiency in handling non‐linear nutritional databases, leading to superior outcomes. Also, the proposed framework resolved the issues faced by the conventional models, such as poor convergence, local optima, struggle in capturing the complex interactions between variables, high computational complexity, etc., leading to enhanced reliability and accuracy in assessing the child's nutritional status.

These improved results highlight the immense advantage of applying an AI‐driven approach to processing large nutritional databases and understanding their patterns for enhanced nutritional assessment research in the country. Furthermore, the proposed model mitigates the manual processing of data and predicts the nutritional status automatically, resulting in cost reduction, minimal computational time, and better accuracy. In addition, the application of the proposed model allows the government to understand the factors influencing child malnutrition and assists them in making policy interventions for reducing malnutrition among children. These insights serve as a foundation for future AI‐driven health assessments, advancing nutritional status classification and predictive modeling in public health research. Moving forward, the study's conclusions offer valuable insights for policymakers, researchers, and healthcare professionals, facilitating evidence‐based decision‐making for childhood malnutrition interventions.

## 5 Conclusion

Addressing childhood malnutrition requires targeted interventions, including improved vaccination programs, enhanced dietary strategies, and optimized public health policies to strengthen children's immune systems and overall well‐being. This study introduces a novel artificial intelligence‐based classification framework, integrating FHO‐K‐Means clustering and EGBF classification to assess the nutritional status of children under five in developing countries. The findings highlight India as the most affected nation among those studied, with household financial conditions significantly influencing childhood malnutrition rates. Additionally, the study reveals that boys' nutritional requirements demand urgent attention to mitigate malnutrition risks effectively. The proposed AI model achieved exceptional performance, with 99.84% accuracy, 99.5% precision, 99.8% specificity, 100% sensitivity, and an F1‐measure of 98.6%, surpassing traditional classification techniques such as RF, XGBT, LR, and ANN. These results underscore the effectiveness of the integration of the FHO‐K‐Means and EGBF model for nutritional status classification, and this AI‐driven framework offers data‐informed decision‐making, facilitating targeted nutritional interventions to combat childhood malnutrition efficiently. Although the presented framework offered improved accuracy, it is not validated across diverse nutritional databases, which limits its scalability and adaptability in real‐time scenarios. Also, the combination of multiple techniques increases the computational burden of the system, particularly while processing large data. Therefore, future studies should concentrate on designing a lightweight AI–based classification system to reduce the computational burden. In addition, future work should explore diverse databases for analyzing the influences of socioeconomic and environmental factors on child malnutrition.

## Author Contributions

All authors contributed equally to conceptualization, methodology, data analysis, writing—original draft, and review and editing.

## Conflicts of Interest

The authors declare no conflicts of interests.

### Peer Review

The peer review history for this article is available at https://publons.com/publon/10.1002/brb3.70548

## Data Availability

All datasets are available at https://www.kaggle.com/c/histopathologic‐cancer‐detection.
